# The Influence of Programmed Cell Death in Myeloid Cells on Host Resilience to Infection with *Legionella pneumophila* or *Streptococcus pyogenes*

**DOI:** 10.1371/journal.ppat.1006032

**Published:** 2016-12-14

**Authors:** Pia Gamradt, Yun Xu, Nina Gratz, Kellyanne Duncan, Lester Kobzik, Sandra Högler, Pavel Kovarik, Thomas Decker, Amanda M. Jamieson

**Affiliations:** 1 Max F. Perutz Laboratories, University of Vienna, Vienna, Austria; 2 CIRI, International Center for Infectiology Research, Université de Lyon, Lyon, France; 3 Inserm U111, Lyon, France; 4 Ecole Normale Supérieure de Lyon, Lyon, France; 5 Université Lyon 1, Centre International de Recherche en Infectiologie, Lyon, France; 6 CNRS, UMR 5308, Lyon, France; 7 Division of Biology and Medicine, Department of Molecular Microbiology and Immunology, Brown University, Providence, Rhode Island, United States; 8 Department of Environmental Health, Harvard School of Public Health, Boston, Massachusetts, United States; 9 Institute of Pathology and Forensic Veterinary Medicine, Department of Pathobiology, University of Veterinary Medicine Vienna, Vienna, Austria; University of Michigan Medical School, UNITED STATES

## Abstract

Pathogen clearance and host resilience/tolerance to infection are both important factors in surviving an infection. Cells of the myeloid lineage play important roles in both of these processes. Neutrophils, monocytes, macrophages, and dendritic cells all have important roles in initiation of the immune response and clearance of bacterial pathogens. If these cells are not properly regulated they can result in excessive inflammation and immunopathology leading to decreased host resilience. Programmed cell death (PCD) is one possible mechanism that myeloid cells may use to prevent excessive inflammation. Myeloid cell subsets play roles in tissue repair, immune response resolution, and maintenance of homeostasis, so excessive PCD may also influence host resilience in this way. In addition, myeloid cell death is one mechanism used to control pathogen replication and dissemination. Many of these functions for PCD have been well defined *in vitro*, but the role *in vivo* is less well understood. We created a mouse that constitutively expresses the pro-survival B-cell lymphoma (bcl)-2 protein in myeloid cells (CD68(bcl2tg), thus decreasing PCD specifically in myeloid cells. Using this mouse model we explored the impact that decreased cell death of these cells has on infection with two different bacterial pathogens, *Legionella pneumophila* and *Streptococcus pyogenes*. Both of these pathogens target multiple cell death pathways in myeloid cells, and the expression of bcl2 resulted in decreased PCD after infection. We examined both pathogen clearance and host resilience and found that myeloid cell death was crucial for host resilience. Surprisingly, the decreased myeloid PCD had minimal impact on pathogen clearance. These data indicate that the most important role of PCD during infection with these bacteria is to minimize inflammation and increase host resilience, not to aid in the clearance or prevent the spread of the pathogen.

## Author Summary

Multicellular organisms are constantly interacting with microbes. Pathogens are microbes that can cause harm to the host if not properly controlled, therefore pathogen clearance is an essential part of survival of all multi-cellular organisms. Equally important factors in survival are host resilience mechanisms, or host processes that increase survival independent of pathogen burden. Not only can pathogens themselves cause damage to the host, the immune response that eradicates pathogens can cause harm in the form of immunopathology. Controlling and repairing damage are important factors in host resilience, and depending on the site of infection the specific mechanisms vary. This study examines the multiple roles that cells of the innate immune response play in both pathogen clearance and host resilience in response to both systemic and pulmonary pathogens.

## Introduction

Pathogen clearance and host resilience/tolerance are both important in surviving a given infection [1,2] [3] [4] [5]. A main purpose of the immune response is to identify and clear invading pathogens. However, highly resilient hosts can survive infection with a given pathogen, independent of the ability of the immune response to clear it. One aspect of host resilience is prevention and repair of extensive tissue damage. Both the immune response and pathogens themselves can cause damage to the infected host [2] [3] [4] [5]. So while the immune system must act to clear a pathogen, it must also be carefully controlled in order to prevent excessive damage. This study seeks to understand the role that myeloid cells, cells of the innate immune response, play in both pathogen clearance and host resilience.

Myeloid cells, including monocytes, macrophages, dendritic cells (DCs), and neutrophils, are an essential part of the innate immune system. During the early stages of the immune response they are essential in both direct phagocytosis and destruction of pathogens, and activation of other immune cells by secretion of cytokines and chemokines [6–9] [10] [8]. They also have important roles in immunoregulation and tissue repair that are crucial in surviving an infection [11–13] [14] [15] [16]. If myeloid cells are not carefully controlled they can cause excessive inflammation that can lead to immunopathology and decreased host resilience [17–20] [10] [11] [21]. The importance of myeloid cells in the innate immune response against infection often makes them a target of pathogens. Microorganisms will manipulate the cells in order to survive, proliferate, and spread to other cells [7,22] [23]. One mechanism of both controlling pathogen replication and host inflammation is programmed cell death (PCD). There are many different PCD pathways of which apoptosis and autophagic cell death are largely non-inflammatory [6,16,24,25] and pyroptosis and necrosis are considered inflammatory [15,22,26] [27]. Apoptotic cell death is controlled by a caspase cascade, of which caspase-3 and caspase-7 are central players [28]. Pro- and anti-apoptotic proteins regulate apoptotic cell death. One key protein that regulates many types of PCD, but in particular apoptosis is B-cell lymphoma (bcl)-2. Infected myeloid cells will undergo cell death in order to control pathogen dissemination and replication [27] [29] [30] [31] [32]. Once the pathogen is cleared myeloid cells will undergo apoptosis to prevent excessive inflammation and immunopathology [24,25] [33] [34] [35] [26].

We have developed a mouse model with decreased myeloid cell death in order to understand the impact it has on pathogen clearance and host resilience to infection. This mouse expresses the anti-apoptotic protein human bcl-2 under the control of the CD68 promoter (CD68(bcl2)tg). This limits ectopic bcl-2 expression to cells of the myeloid lineage including monocytes, macrophages, neutrophils, and DCs. Bcl-2 primarily prevents apoptotic and autophagic cell death [25] [36] [37,38], thus making this an ideal model for studying the role of non-inflammatory myeloid PCD in pathogen clearance and host resilience.

This study uses two bacterial pathogens, *L*. *pneumophila* and *S*. *pyogenes*, that infect distinct sites, to examine how decreasing myeloid cell death impacts pathogen clearance and host resilience. While many studies have demonstrated mechanisms of myeloid cell death in *in vitro* infection models of *S*. *pyogenes and L*. *pneumophila* [39–44] [33], it remains unclear what role myeloid cell death plays during *in vivo* infection. *L*. *pneumophila* infection remains confined to the lung under most circumstances where it causes a severe pneumonia [45] [46]. This bacteria is found in contaminated water supplies, such as air-conditioning systems, and infects alveolar macrophages [45,47,48] [46]. It can cause complications in people with immunosuppression or other health problems, making it an important hospital-acquired infection [49] [50]. In mice, pulmonary infection can be mimicked using an intranasal infection model of *L*. *pneumophila*. *S*. *pyogenes* is a versatile pathogen that infects many areas of the body including the upper respiratory tract and soft tissue [51]. Invasive soft tissue infections can result in the systemic spread of bacteria causing a severe toxic shock syndrome (TSS) [35] [50] [29] [52]. To mimic this type of infection, we used a cutaneous infection model that rapidly causes a systemic infection. Using these two models we examined the roles that myeloid cell death play during both pulmonary and systemic infections.

*L*. *pneumophila* primarily infects lung macrophages, and actively delays apoptosis of these cells in order to replicate [53] [54] [55] [56] [31]. Infection with *L*. *pneumophila* induces an early pyroptotic cell death under the control of caspase-1 [57,58] [59] [60] [43] [61] [40] [62] [42]. There is also a caspase-11-dependent cell death that has shown *in vitro* to be independent of flagellin [40,57]. The later apoptotic cell death is at least partly also under the control of caspase-3, and as such can be inhibited by bcl-2 [63] [64]. Human macrophages do not express the Naip5 inflammasome that is triggered by *L*. *pneumophila* flagellin, so to better mimic the human infection we use a strain of *L*. *pneumophila* lacking flagellin A (ΔflaA). Deletion or inhibition of the pro-survival factor BCL-XL in macrophages results in decreased *L*. *pneumophila* replication [65], indicating that delaying PCD is a strategy that *L*. *pneumophila* may have for surviving in cells. When macrophages eventually undergo apoptosis this may enable the pathogen to spread to other cells. Unlike macrophages, DCs do not support the growth of *L*. *pneumophila* as they undergo rapid cell death in response to infection. When apoptotic cell death is blocked in DCs by overexpression of bcl-2 *L*. *pneumophila* will proliferate in DCs [27]. It was hypothesized that since DCs migrate throughout the body this DC cell death may be a mechanism to prevent spread of the bacteria.

Similar to *L*. *pneumophila*, *S*. *pyogenes* is thought to cause PCD by pyroptosis and apoptosis [29] [66]. The role that this PCD plays during infection is not well understood. The severe inflammatory response caused by *S*. *pyogenes* infection may be tempered by PCD in myeloid cells such as macrophages and neutrophils [67] [35] [68] [69]. *S*. *pyogenes* causes lysis of myeloid cells in a streptolysin O-dependent manner, that is thought to increase pathogen spread [68] [29] [52]. The PCD induced by *S*. *pyogenes* could be an immune evasion technique, and strains that cause less PCD have reduced virulence [29]. Therefore myeloid PCD may impact both pathogen clearance and host resilience to *S*. *pyogenes* infection.

This study explores *in vivo* the role that myeloid PCD plays during infection with two distinct pathogens. While the role of PCD in response to infection is well documented *in vitro*, less is known about what sort of balance is struck between controlling pathogen clearance and maintaining host resilience during *in vivo* infections. Both of the bacterial pathogens used in this study interact with myeloid cell death pathways, and this study focuses on the role that cell death controlled by bcl-2 plays during infection. Our data demonstrates that CD68(bcl-2)tg mice infected with either pathogen have decreased host resilience that occurs largely independent of any changes in pathogen clearance. This indicates that the rate of myeloid cell death is calibrated to preserve host resilience, and manipulations of this rate are detrimental to the host.

## Results

### Infection with *L*. *pneumophila* and *S*. *pyogenes* induces apoptotic cell death in macrophages

**Fig 1:**
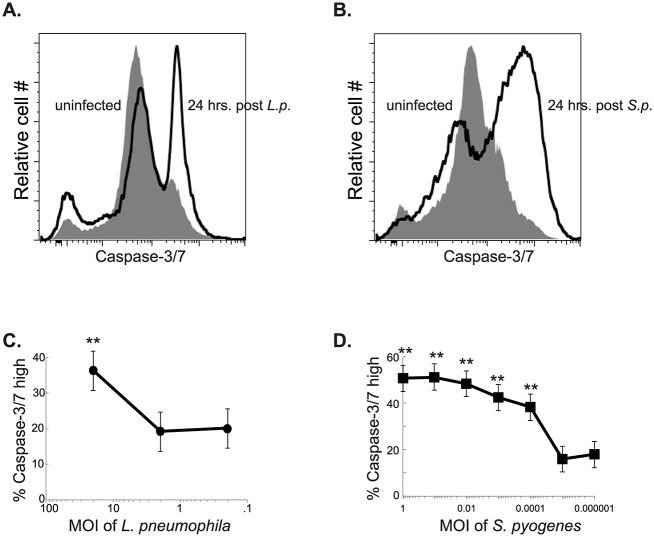
Infection of macrophages with *L*. *pneumophila* or *S*. *pyogenes* results in increased apoptotic cell death. Measurement of active caspase-3/7, using cell event reagent in BMDMs infected with *L*. *pneumophila* (A) or *S*. *pyogenes* (B). Percent of caspase-3/7 BMDMs at titrated MOIs of *L*. *pneumophila* (C) or *S*. *pyogenes* (D). Data shown is representative of at least 3 independent experiments with n = 3–4 per experiment. The mean values are displayed. * denotes P value ≤ 0.05. ** denotes P value ≤ 0.001.

Bone marrow derived macrophages (BMDM) were infected with *L*. *pneumophila*. Human macrophages are more permissive to infection by *L*. *pneumophila* than macrophages derived from most common mouse strains [70]. Triggering of the NAIP5 inflammasome by flagellin, results in rapid pathogen clearance in mouse macrophages. In order to better recapitulate the infection that is seen in human macrophages for our mouse model we used a ΔflaA strain of *L*. *pneumophila*. To compensate for the lack of motility the bacteria are spun briefly onto cells. Twenty-four hours after infection with *L*. *pneumophila* there was an increased number of apoptotic cells as indicated by flow cytometry staining using a stain for activated caspase-3/7. (Fig 1A). Similarly there was an increase in caspase-3/7 activation in BMDMs infected with *S*. *pyogenes* (Fig 1B). This apoptosis was dependent on the dose of bacteria given. *L*. *pneumophila* induced the most cell death at a multiplicity of infection (MOI) of 20 (Fig 1C). *S*. *pyogenes* induced cell death at an MOI as low as .001 (Fig 1D).

### Expression of bcl-2 under the control of the CD68 promoter prevents macrophage cell death

**Fig 2:**
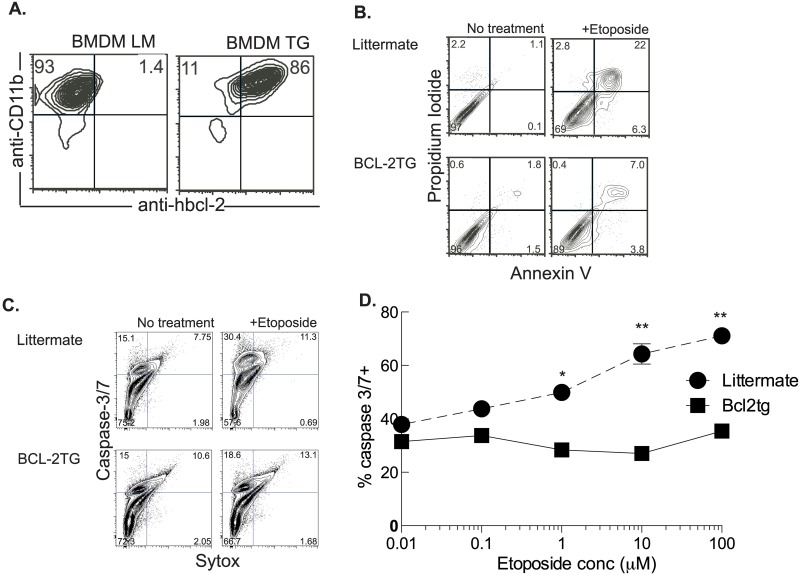
Overexpression of bcl-2 rescues macrophages from etoposide-induced cell death. Detection of human bcl-2 by intracellular staining in BMDMs derived from CD68(bcl2)tg mice and littermate controls (A). Etoposide-induced cell death in BMDMs derived from transgenic and littermate controls is detected by staining with annexin V and propidium iodide (B). The induction of apoptosis by etoposide in transgenic and littermate BMDMs is further confirmed by detection of activated caspase-3/7 and staining with sytox for detecting membrane permeability (C). Etoposide dose-dependent activation of caspases in BMDMs from littermate and CD68(bcl2)tg mice (D). Staining is representative of at least 3 independent experiments with n = 3–4 per experiment. The mean values are displayed. * denotes P value ≤ 0.05. ** denotes P value ≤ 0.001.

Macrophages were derived from mice expressing human bcl-2 under the control of the CD68 promoter. Most of these macrophages constitutively express bcl-2 (Fig 2A). When BMDMs are exposed to the DNA damaging agent etoposide there is an increase in apoptotic cells as demonstrated by staining with annexin V and propidium iodide and flow cytometry analysis. Macrophages constitutively expressing bcl-2 had significantly decreased apoptotic cell death after exposure to etoposide (Fig 2B). As further evidence that ectopic expression of bcl-2 prevents etoposide-induced apoptosis cells were stained with the cell event reagent that is activated by activated caspase-3/7, and the DNA dye sytox that indicates permeable cells. The substantial caspase-3/7 activation induced by etoposide was abrogated in BMDMs derived from CD68(bcl2)tg mice (Fig 2C). The ectopic expression of bcl2 was effective in decreasing apoptosis at both high and low doses of etoposide (Fig 2D).

**Fig 3:**
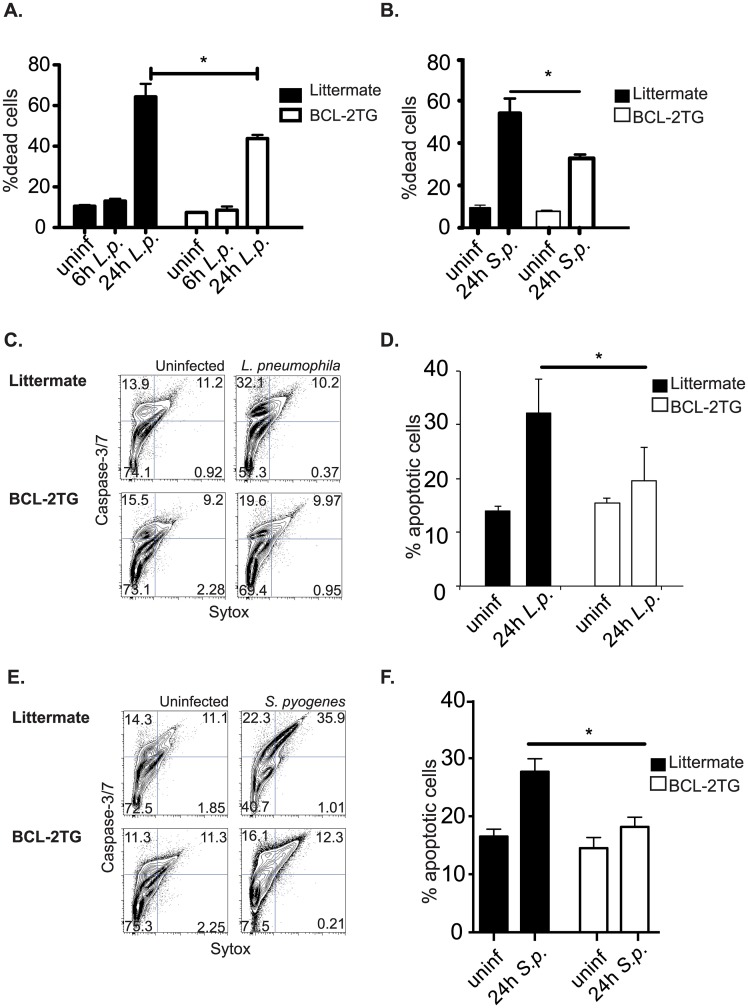
Overexpression of bcl-2 rescues macrophages from bacterial pathogen-induced cell death. Using a fixable viability dye the percent of dead transgenic or littermate BMDMs was measured after infection with *L*. *pneumophila* (A) or *S*. *pyogenes* (B). The type of cell death as further characterized by measurement of active caspase-3/7 and cell permeability using the cell event reagent and sytox respectively. Representative staining of transgenic and littermate BMDMs infected with *L*. *pneumophila* is shown in (C), and quantified in (D). Representative staining of transgenic and littermate BMDMs infected with *S*. *pyogenes* is shown in (E), and quantified in (F). Data shown are representative of at least 3 independent experiments with n = 3–4 per experiment. The mean values are displayed. * denotes P value ≤ 0.05.

Constitutive expression of bcl-2 also decreased the cell death in BMDMs infected with *L*. *pneumophila* (Fig 3A), and *S*. *pyogenes* (Fig 3B) when examined with a fixable live/dead stain 24 hours after infection. The cell death prevented by bcl-2 during infection was largely apoptotic, as indicated by caspase-3/7 activation and cell permeability. Macrophages derived from CD68(bcl2)tg mice, infected for 24 hours with *L*. *pneumophila*, had decreased caspase-3/7 activation compared to macrophages derived from their littermate controls. This is shown by flow cytometry staining (Fig 3C), and quantified (Fig 3D). Likewise flow cytometry performed on macrophages infected with *S*. *pyogenes* indicated that both apoptotic and late apoptotic/necrotic stages were inhibited by ectopic expression of bcl-2 (Fig 3E and 3F).

### Bcl-2 under the control of the CD68 is expressed in macrophages, neutrophils, and dendritic cells

**Fig 4:**
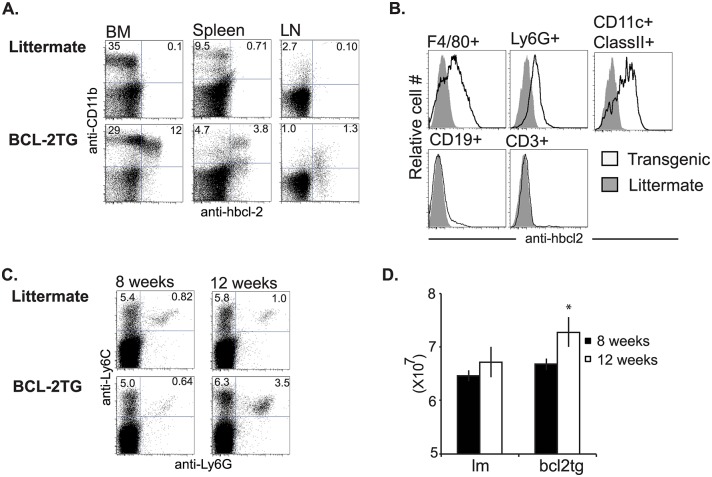
Expression of transgenic bcl-2 in myeloid cell subsets. *In vivo* transgenic bcl-2 is expressed primarily in CD11b+ cells, as shown by intracellular staining for hbcl-2. Cells from in the bone marrow (BM), spleen, and lymph nodes (LN) of the CD68(bcl2)tg mice were examined for expression and compared to littermate controls (A). Cells were further examined by use of cell surface markers and subsets of F4/80+ macrophages, Ly-6G+ neutrophils, CD11c+ClassII+ DCs, CD19+ B cells, and CD3+ T cells (B). Percentage of Ly6C+ and Ly6G+ cells in the spleen of 8 week and 12 week old CD68(bcl2)tg mice compared to littermate controls (C). Number of total cells in the spleens of 8 week and 12 week old CD68(bcl2)tg and littermate control mice (D). These data are representative of at least 3 independent experiments with n = 3–4 per experiment. The mean values are displayed. * denotes P value ≤ 0.05.

Bone marrow, spleen and lymph nodes were examined for expression of transgenic bcl-2. The transgene was expressed primarily in CD11b+ cells in these organs (Fig 4A).

While the transgene is expressed in all myeloid cell subsets it is expressed at the highest level in F4/80+CD11b+ macrophages and lower in Ly6G+ neutrophils (Fig 4B). It is expressed at an intermediate level in CD11c+ MHC class II+ DCs, and not expressed in cells of the lymphocyte lineage (Fig 4B). In 12 week-old mice there is a slight increase in Ly6G+Ly6Clow neutrophils and Ly6G-Ly6Chigh inflammatory monocytes, while in mice aged 6–8 weeks the myeloid compartments are comparatively normal (Fig 4C). These changes are most noticeable in the spleens of older mice (Fig 4C). The spleens of 12 week old mice were slightly larger than their littermate controls (Fig 4D), therefore the total number of inflammatory monocytes and neutrophils was also higher. The spleen cellularity was comparable between CD68(bcl2)tg mice and littermate controls at 8 weeks. There was no indication of cancer development as the mice aged. The mice were healthy and lived a normal lifespan. However, given this slight accumulation of inflammatory cells as mice aged we used mice that were between 6–8 weeks of age for the infection experiments. This enabled us to focus primarily on the impact that decreased myeloid cell death had on infection, and not on homeostatic effects of constitutive bcl-2 expression.

### Expression of bcl-2 under the control of the CD68 promoter prevents cell death in myeloid cell subsets

**Fig 5:**
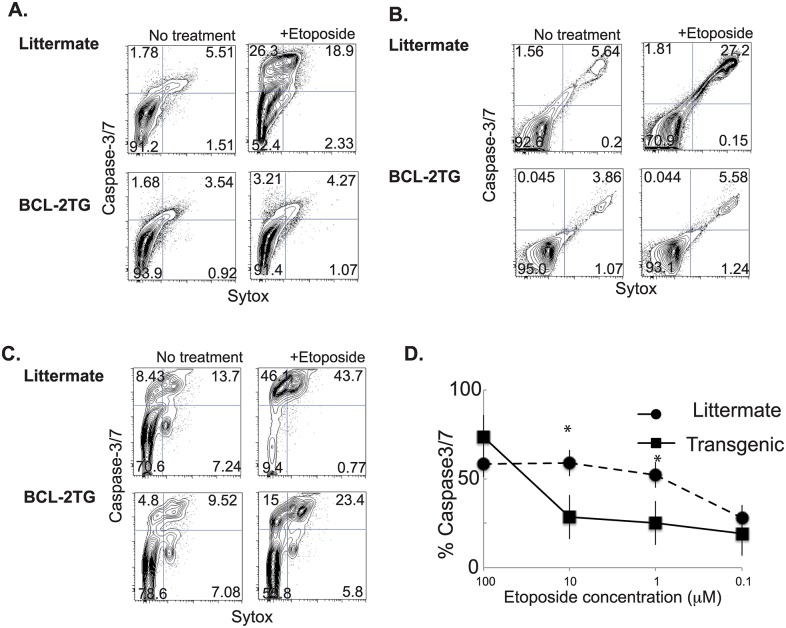
Overexpression of bcl-2 rescues other myeloid cell types from etoposide-induced cell death. Effect of bcl-2 transgene on etoposide-induced caspase-3/7 activation in bone marrow derived DCs (BMDC) (A), neutrophils (B), and resident peritoneal macrophages (C), as measured by staining of activated caspase-3/7 and sytox. Peritoneal apoptosis is quantified in (D). These data are representative of at least 3 independent experiments with n = 3–4 per experiment. The mean values are displayed. * denotes P value ≤ 0.05.

Since the expression of bcl2 is expressed at varying levels in the different myeloid cell types, we examined the ability of the transgene to rescue DCs, neutrophils, and resident peritoneal macrophages from etoposide-induced cell death. Bone marrow-derived DCs (BMDCs) had a large increase in caspase-3/7 activation after treatment with etoposide, but this was greatly decreased with the presence of bcl-2 (Fig 5A). Neutrophils had an increase in late apoptotic or necrotic cells after treatment with etoposide based on staining for caspase-3/7 and sytox (Fig 5B). This PCD was also prevented by the ectopic expression of bcl-2 (Fig 5B). To show the effect of bcl-2 on a different subset of macrophages we used resident peritoneal macrophages. Etoposide-induced apoptosis and necrosis was decreased in peritoneal macrophages isolated from CD68(bcl2)tg mice, compared to littermate controls (Fig 5C and 5D).

### Impact of decreased myeloid cell death during infection with *L*. *pneumophila* on host resilience and pathogen clearance

**Fig 6:**
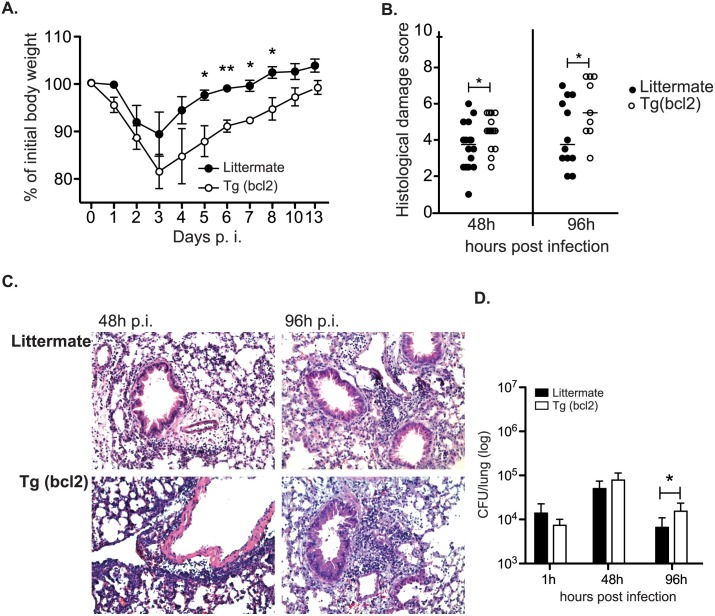
Effect of ectopic bcl-2 expression on health, lung damage, and pulmonary CFUs after infection with *L*. *pneumophila*. Weight loss and recovery in CD68(bcl2)tg mice and littermate controls after infection with 1X10^6^ *L*. *pneumophila* (A). The histological damage score (HDS) in CD68(bcl2)tg mice and littermate controls 48 hours and 96 hours after infection with *L*. *pneumophila* (B). Representative lung histology samples stained with H and E from CD68(bcl2)tg mice 48 hours and 96 hours after infection with *L*. *pneumophila* (C). Bacterial CFUs in the lung of CD68(bcl2)tg mice 1, 48, and 96 hours after infection with *L*. *pneumophila* as compared to littermate controls (D). These data are representative of at least 3 independent experiments with n = 3–4 per experiment. The mean values are displayed. * denotes P value ≤ 0.05. ** denotes P value ≤ 0.001.

In order to determine the impact that decreased myeloid cell death had on pathogen clearance and host resilience responses during pulmonary infection, mice were infected with the bacterial pathogen *L*. *pneumophila*. Mice infected intranasally with 1X10^6^ *L*. *pneumophila* start to steadily lose weight 2 days after infection (Fig 6A). After infection CD68(bcl2)tg mice lose more weight and have a longer recovery time when compared to littermate controls (Fig 6A).

We next examined the specific cause of this decreased health status in infected CD68(bcl2)tg mice. One possibility was that the increased survival of myeloid cells, in particular DCs aided in the systemic spread of the bacteria. However, the infection was confined to the lung and there were no detectable bacteria in the spleen, liver or kidneys of infected mice (S1A Fig).

The decreased health of the CD68(bcl2)tg mice was therefore due to activity and responses in the lung. CD68(bcl2)tg mice infected with *L*. *pneumophila* had significantly increased damage in their lungs compared to littermate controls as soon as 48 hours after infection and this damage was even greater 96 hours after infection in transgenic mice (Fig 6B and 6C). The histological damage score (HDS) measured a number of parameters including area of damage and immune cell infiltration into the alveolar space. In order to determine if this increased lung damage was due to an increased bacterial burden in the lung of CD68(bcl2)tg mice we determined the colony forming units (CFUs) in the lungs from infected mice 1 hours, 48 hours, and 96 hours after infection. There was no statistically significant difference between genotypes in bacterial uptake in the lung or early proliferation as indicated by the bacterial counts at 1 hour after infection and 48 hours after infection. Interestingly, the lung damage preceded the small increase in bacteria counts in transgenic mice observed 96 hours after infection (Fig 6D). Ten days after infection both transgenic mice and littermate controls had cleared the infection (S1B Fig).

### CD68(bcl2)tg have increased cellularity, cytokine, and chemokine expression compared to littermate controls after infection with *L*. *pneumophila*

**Fig 7:**
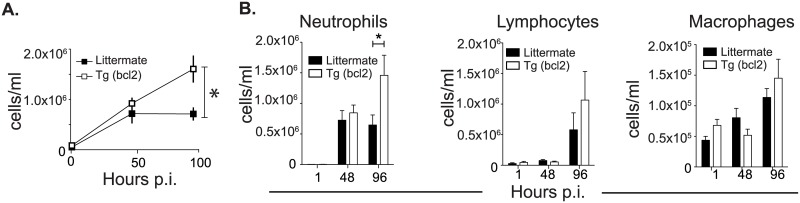
Cells infiltrate into lungs of *L*. *pneumophila* infected mice. Cellular infiltrate in to the lungs of CD68(bcl2)tg mice and littermate controls 48 hours and 96 hours after infection with 1X10^6^ *L*. *pneumophila*. Cells are counted from 1 ml of the bronchoalveolar lavage fluid (BALF) from each mouse (A). Identification of neutrophils, lymphocytes, and macrophages in the BALF after cytospin analysis (B). These data are representative of at least 3 independent experiments with n = 3–4 per experiment. The mean values are displayed. * denotes P value ≤ 0.05.

In order to determine the cause of the increased inflammation and lung damage, the pulmonary immune response to *L*. *pneumophila* was analyzed in CD68(bcl2)tg mice and littermate controls. Both transgenic mice and littermate controls had the same amount of immune cell infiltrate in the bronchoalveolar lavage fluid (BALF) 48 hours after infection (Fig 7A).

The number of infiltrating cells remained about the same in littermates 96 hours after infection, but continued to increase in the lungs of transgenic animals (Fig 7A). The increased infiltrating immune cells in transgenic mice were mostly neutrophils as determined by cytospin analysis (Fig 7B). Neutrophils migrated into the lungs of infected animals by 48 hours after infection, and their number was increased in transgenic animals compared to littermate controls 96 hours after infection. Surprisingly, other cell types had no significant increase in the lungs of infected CD68(bcl2)tg mice compared to infected littermate controls (Fig 7B). Macrophages increased in the lungs of both CD68(bcl2)tg mice and littermate controls at the same rate (Fig 7B).

**Fig 8:**
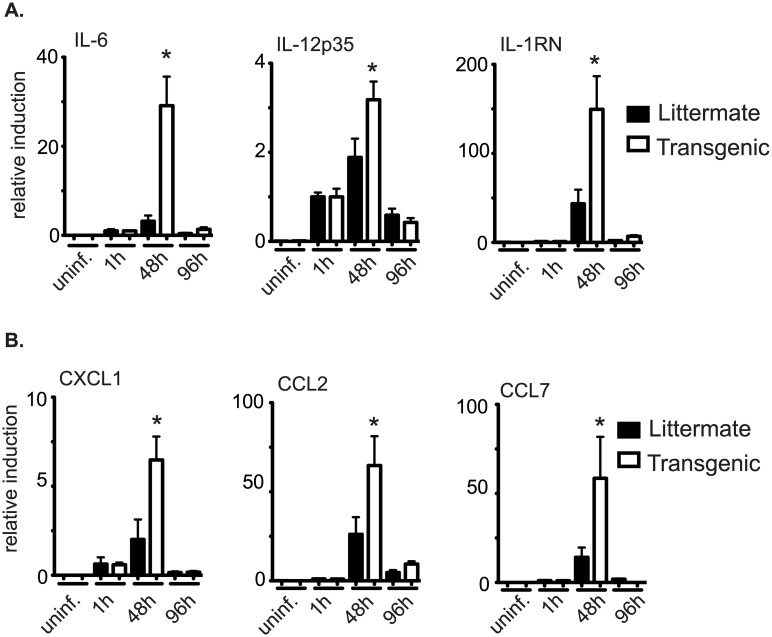
Cytokines and chemokines in lungs of mice infected with *L*. *pneumophila*. Expression of cytokines (A) and chemokines (B) in the lungs of CD68(bcl2)tg mice compared to littermate controls as measured by qPCR. These data are representative of at least 3 independent experiments with n = 3–4 per experiment. The mean values are displayed. * denotes P value ≤ 0.05.

In addition to looking at the immune cell infiltrate, expression levels of several cytokines and chemokines were measured in the lungs of infected animals (Fig 8A and 8B).

The peak of expression of these inflammatory genes in the lungs of both transgenic mice and littermate controls was 48 hours after infection, but the expression of several cytokines and chemokines were elevated in transgenic animals compared to littermate controls (Fig 8A and 8B). The large increase of IL-6 indicates an increase in inflammation in the lungs of these animals [71]. IL-1 receptor antagonist (*Il-1rn*) expression is also increased 48 hours after infection in transgenic animals. This gene is known to be important in resolution of lung inflammation and the expression is increased during times of acute inflammation [72] Also CXCL1 and CCL7 are known chemoattractants for neutrophils [73] and the increased expression of these chemokines at 48 hours may lead to the increase recruitment of the neutrophils by 96 hours. While there is a mild increase in bacterial load in lungs of infected CD68(bcl2)tg mice 96 hours after infection, the increased inflammation precedes this increase in bacterial burden. Therefore it seems that decreased myeloid cell death impacts host resilience, but does not affect the spread of the bacterial pathogen, and only slightly delays the clearance.

### Constitutive bcl2 expression in multiple myeloid cell subsets prevents *L*. *pneumophila*-induced cell death, but does not impact cytokine production

**Fig 9:**
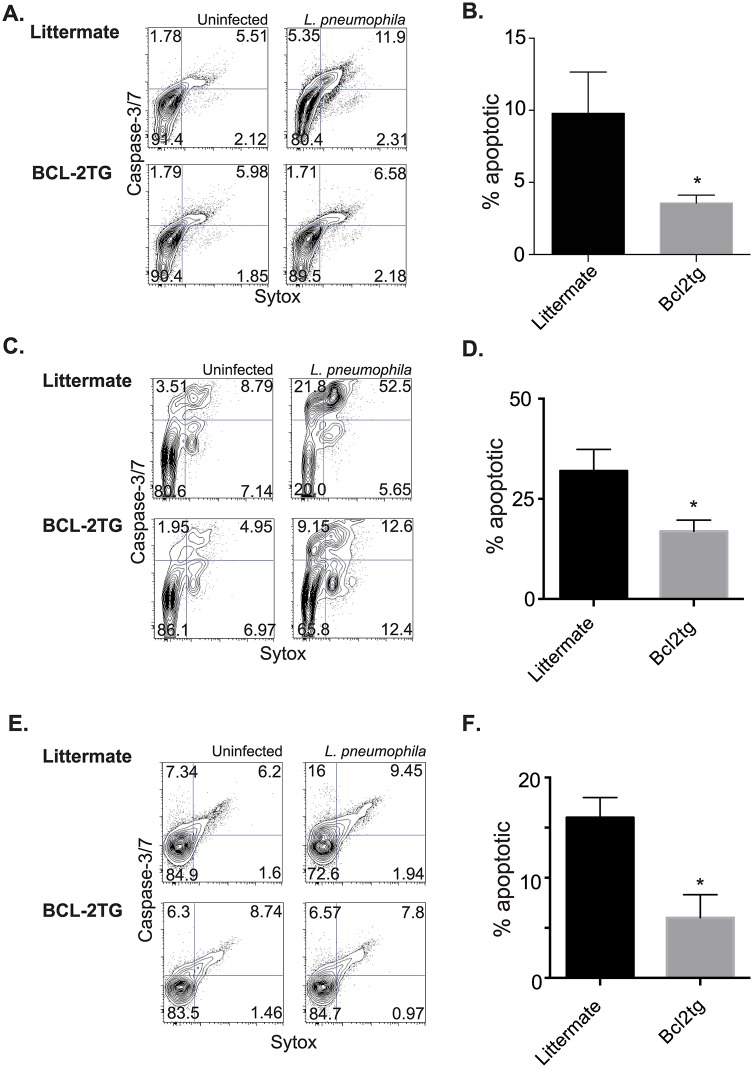
Overexpression of bcl-2 rescues other myeloid cell types from *L*. *pneumophila*-induced cell death. Caspase-activation is measured in BMDC from CD68(bcl2)tg mice and littermate controls after infection with *L*. *pneumophila*. A representative staining is shown in (A), and the amount of apoptotic cells is quantified in (B). Caspase-activation is measured in alveolar macrophages from CD68(bcl2)tg mice and littermate controls after infection with *L*. *pneumophila*. A representative staining is shown in (C), and the amount of apoptotic cells is quantified in (D). Caspase-activation is measured in neutrophils from CD68(bcl2)tg mice and littermate controls after infection with *L*. *pneumophila*. A representative staining is shown in (E), and the amount of apoptotic cells is quantified in (F). These data are representative of at least 3 independent experiments with n = 3–4 per experiment. The mean values are displayed. * denotes P value ≤ 0.05.

Given the impact that the CD68(bcl2)tg has on *in vivo* infection with *L*. *pneumophila*, we explored how *L*. *pneumophila*-induced PCD is affected by expression of bcl2 in relevant myeloid cell types. DCs from CD68(bcl2)tg mice had decreased PCD after infected with *L*. *pneumophila*, compared to littermate controls, as demonstrated with caspase-3/7 activation and cell permeability assays (Fig 9A and 9B). Alveolar macrophages isolated from CD68(bcl2)tg mice also had decreased *L*. *pneumophila*-induced early and late apoptosis compared to littermate controls (Fig 9C and 9D). While there is limited detection of PCD in neutrophils infected with *L*. *pneumophila*, the small amount of apoptosis observed is rescued by ectopic expression of bcl-2 (Fig 9E and 9F).

**Fig 10:**
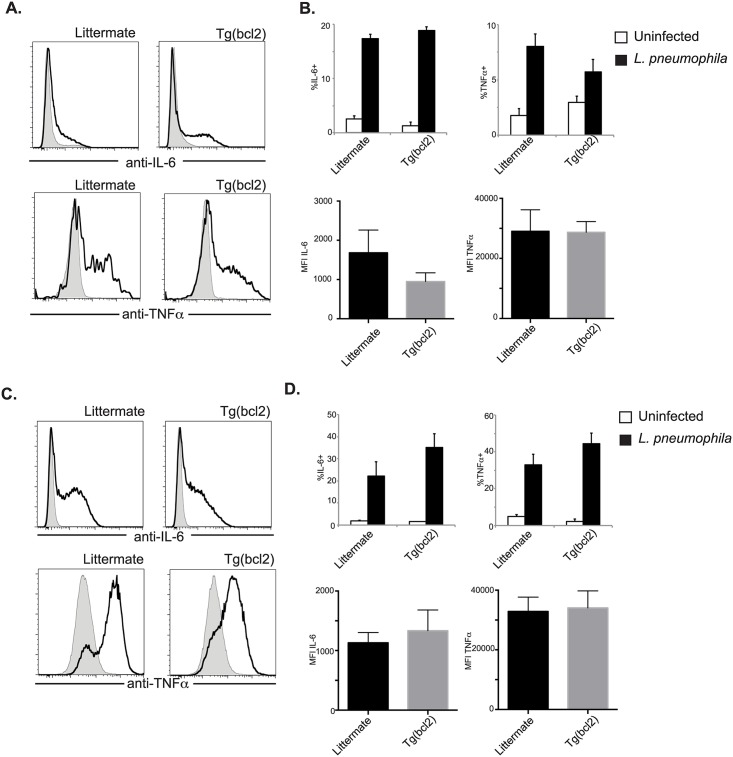
Cytokine production from DCs and macrophages after *L*. *pneumophila* infection. Production of IL-6 and TNF**α** was measured by intracellular staining. Grey shaded histograms are uninfected and *L*. *pneumophila* infected are depicted with solid black lines. Representative staining of DCs is shown in (A), the percent of cells expressing cytokines is shown in (B top), and the MFI is shown in (B bottom). Representative staining of macrophages is shown in (C), the percent of cells expressing cytokines is shown in (D top), and the MFI is shown in (D bottom). These data are representative of at least 3 independent experiments with n = 3–4 per experiment. The mean values are displayed. * denotes P value < .05.

During infection with *L*. *pneumophila* there is an increase in cytokines detected in the lung. To investigate if this is caused by an increase of cells producing cytokines, or if the transgenic myeloid cells make more cytokines, infected DCs and macrophages were stained intracellularly for IL-6 and TNFα. There was not a significant increase in the percentage of DCs making either cytokine when CD68(bcl2)tg mice were compared to littermate controls infected with *L*. *pneumophila* (Fig 10A and 10B (top)). Also the mean fluorescent intensity was the same in DCs from transgenic animals or littermate controls (Fig 10B (bottom)), indicating that on a per cell basis the cytokine production is the same. Similar results were observed for macrophages. Cytokine production was equivalent between macrophages from transgenic mice and littermate controls, on a per cell and population basis (Fig 10C and 10D). We interpret this to mean that the increase in cytokines observed in the lungs of CD68(bcl2)tg mice is due to the increased number of inflammatory cells.

### Decreased myeloid cell death during infection with *S*. *pyogenes* impacts resilience but not resistance

**Fig 11:**
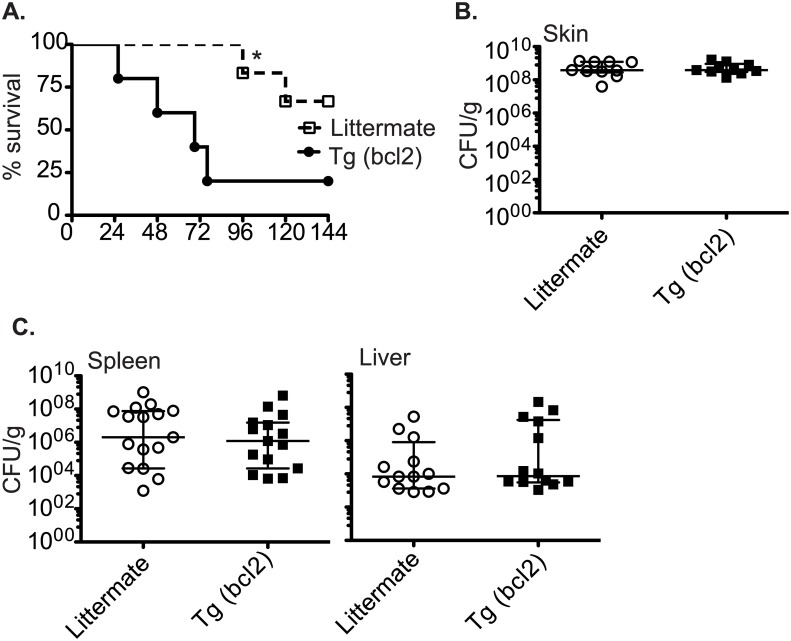
Survival and bacterial load in mice infected with *S*. *pyogenes*. Survival of CD68(bcl2)tg mice compared to littermate controls after subcutaneous infection with 1X10^7^ *S*. *pyogenes* (A). Bacterial load in CD68(bcl2)tg mice compared to littermate controls at the site of infection as measured by CFU 2 days after infection. (B). Bacterial load in CD68(bcl2)tg mice compared to littermate controls in the livers and spleens as measured by CFU 2 days after infection. (C). Data shown represent 4 experiments with n = 3–4 per experiment. The median CFUS are displayed. * denotes P value < .05.

In order to determine how decreased myeloid cell death would impact the response to a systemic pathogen we infected CD68(bcl2)tg mice and littermate controls with *S*. *pyogenes*. In order to mimic a common course of severe infection with *S*. *pyogenes*, a cutaneous infection that causes systemic disease, the bacteria were injected subcutaneously. The bacteria spread from the skin into the blood stream within a few hours and colonize various organs. Mice constitutively expressing bcl-2 in myeloid cells had decreased survival after infection with *S*. *pyogenes (*Fig 11A).

However, there was no statistically significant change in the bacterial load in these mice (Fig 11B). The initial site of infection, the skin, had similar bacterial loads between transgenic mice and littermate controls 2 days after infection. In addition, the systemic spread was also similar as the spleen, and the liver had similar levels between the two genotypes (Fig 11C).

**Fig 12:**
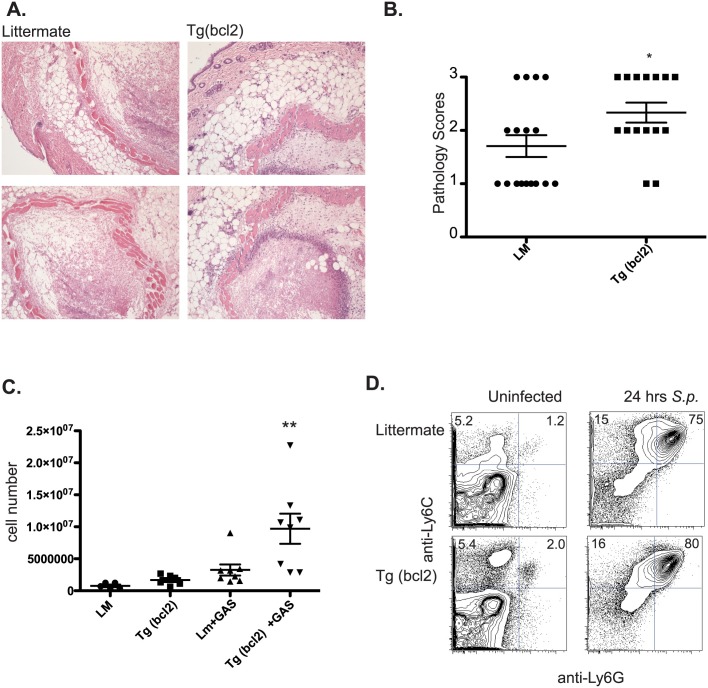
Immune cell infiltrate after infection with *S*. *pyogenes*. Representative histology micrographs of infected skin from CD68(bcl2)tg mice and littermate controls from mice infected with *S*. *pyogenes* 2 days after infection (A). Pathology scores of skin from CD68(bcl2)tg mice and littermate controls 2 days after infection with *S*. *pyogenes* (B). Cell numbers in the peritoneal cavity of CD68(bcl2)tg mice and littermate controls 24 hours after injection of heat-killed *S*. *pyogenes* into the peritoneal cavity compared with uninfected controls (C). Flow cytometry staining of Ly6C+ and Ly6G+ cells in the peritoneal cavity of CD68(bcl2)tg mice and littermate controls before and after infection with *S*. *pyogenes*. These data are representative of at least 3 independent experiments with n = 3–4 per experiment. The mean values are displayed. * denotes P value ≤ 0.05. ** denotes P value ≤ 0.001.

The decreased survival despite the unchanged pathogen clearance rates between the genotypes indicated a decrease in host resilience, and we examined a number of factors that could contribute to this. We examined the site of infection to determine if there were changes in inflammatory immune cell infiltration. There were increased infiltrating cells in transgenic mice into the area of bacterial infection (Fig 12A and 12B).

To get a clearer understanding of the types of immune cells infiltrating into the site of infection *S*. *pyogenes* was injected intraperitoneally. There was a significant increase in infiltrating cells into the peritoneal cavity of CD68(bcl2)tg mice 24 hours after infection with *S*. *pyogenes* (Fig 12C). However, the types of cells that responded to the infection did not change between the littermate and transgenic mice (Fig 12D). The responding cells were primarily neutrophils as identified by Ly6G and Ly6C expression in both genotypes of mice (Fig 12D), however the transgenic mice had more cells (Fig 12C).

### Constitutive bcl2 expression prevents *S*. *pyogenes*-induced cell death in multiple myeloid cell subsets

**Fig 13:**
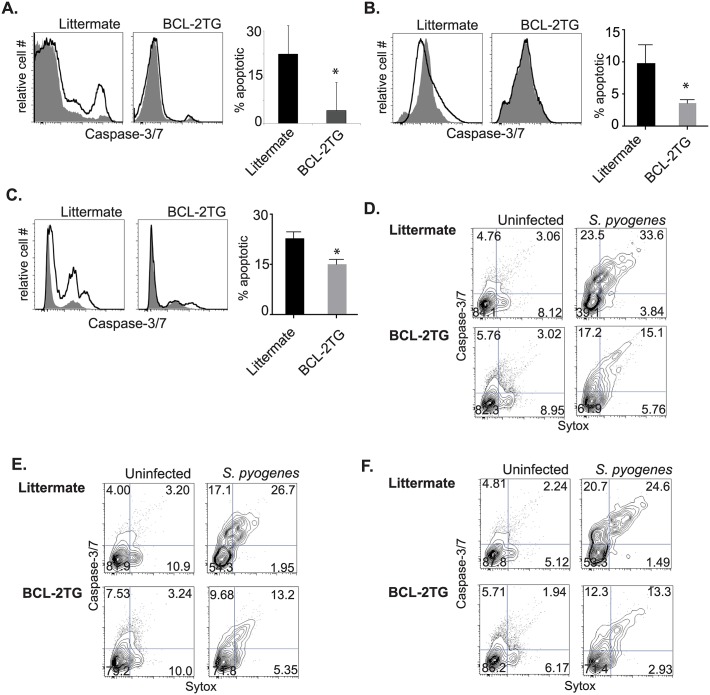
Overexpression of bcl-2 rescues other myeloid cell types from *S*. *pyogenes*-induced cell death. Caspase-activation is measured in neutrophils from CD68(bcl2)tg mice and littermate controls after infection with *S*. *pyogenes*. A representative staining is shown in (A, left), and the amount of apoptotic cells is quantified (A, right). Caspase-activation is measured in BMDC from CD68(bcl2)tg mice and littermate controls after infection with *S*. *pyogenes*. A representative staining is shown in (B, left), and the amount of apoptotic cells is quantified in (B, right). Caspase-activation is measured in resident peritoneal macrophages from CD68(bcl2)tg mice and littermate controls after infection with *S*. *pyogenes*. A representative staining is shown in (C, left), and the amount of apoptotic cells is quantified in (C, right). Ex vivo stains of blood from mice infected with *S*. *pyogenes* was used to look at apoptosis of neutrophils (D), monocytes (E), and DCs (F). These data are representative of at least 3 independent experiments with n = 3–4 per experiment. The mean values are displayed. * denotes P value < .05.

Given the increase in different myeloid cell types during infection with *S*. *pyogenes* in CD68(bcl2)tg mice, the effect of ectopic expression of bcl2 on *S*. *pyogenes*-induced cell death was examined. Neutrophils (Fig 13A), DCs (Fig 13B), and resident peritoneal macrophages (Fig 13C) from CD68(bcl2)tg mice all had significantly decreased *S*. *pyogenes*-induced caspase-3/7 activation. During *in vivo* infection apoptotic cells are rapidly cleared [74], also the processes involved in cell isolation often cause cell death, therefore detection of apoptosis from *ex vivo* samples can be challenging. However, rapid assaying of whole blood cells from mice infected with *S*. *pyogenes* allows for the detection of cells undergoing PCD. *S*. *pyogenes* induces more than half of neutrophils in the blood to undergo PCD, but in CD68(bcl2)tg mice significantly less cells were undergoing PCD (Fig 13D). The same is true for blood monocytes (Fig 13E), and DCs (Fig 13F).

### Constitutive expression of bcl2 in myeloid cells leads to increased systemic cytokine production and liver damage

**Fig 14:**
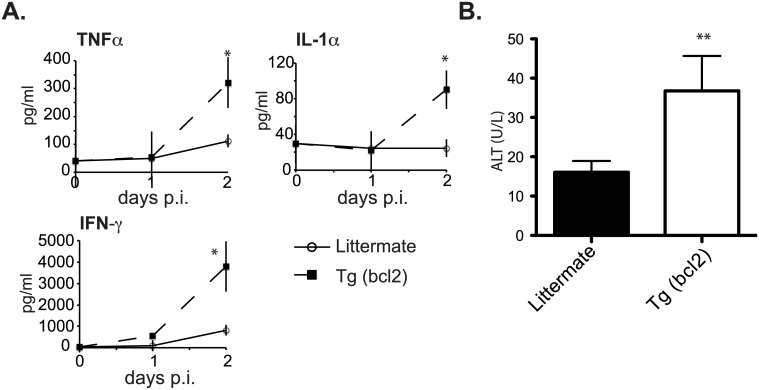
Systemic cytokines and liver damage in mice infected with *S*. *pyogenes*. Serum levels of the inflammatory cytokines TNFα, IL-1α, and IFN-γ 24 and 48 hours after infection in CD68(bcl2)tg mice and littermate controls (A). Serum levels of the liver enzyme ALT in CD68(bcl2)tg mice and littermate controls 48 hours after infection with *S*. *pyogenes* (B). These data are representative of at least 3 independent experiments with n = 3–4 per experiment. The mean values are displayed. * denotes P value ≤ 0.05. ** denotes P value ≤ 0.001.

Since death caused by systemic infection with *S*. *pyogenes* is due to a toxic shock syndrome induced by systemic inflammation we examined the systemic responses. There were many systemic changes during infection. Most notably there were significant changes in the inflammatory cytokines TNFα, IL-1α, and IFN-γ (Fig 14A).

Transgenic mice sustained significant liver damage after infection as evidenced by increased levels of alanine aminotransferase (ALT) in the serum (Fig 14B). The decreased myeloid cell death in this systemic infection resulted in increased systemic inflammation that exacerbated the disease progression and led to decreased host resilience.

### Constitutive bcl2 expression does not lead to increased cytokine production in myeloid cells during *S*. *pyogenes* infection

**Fig 15:**
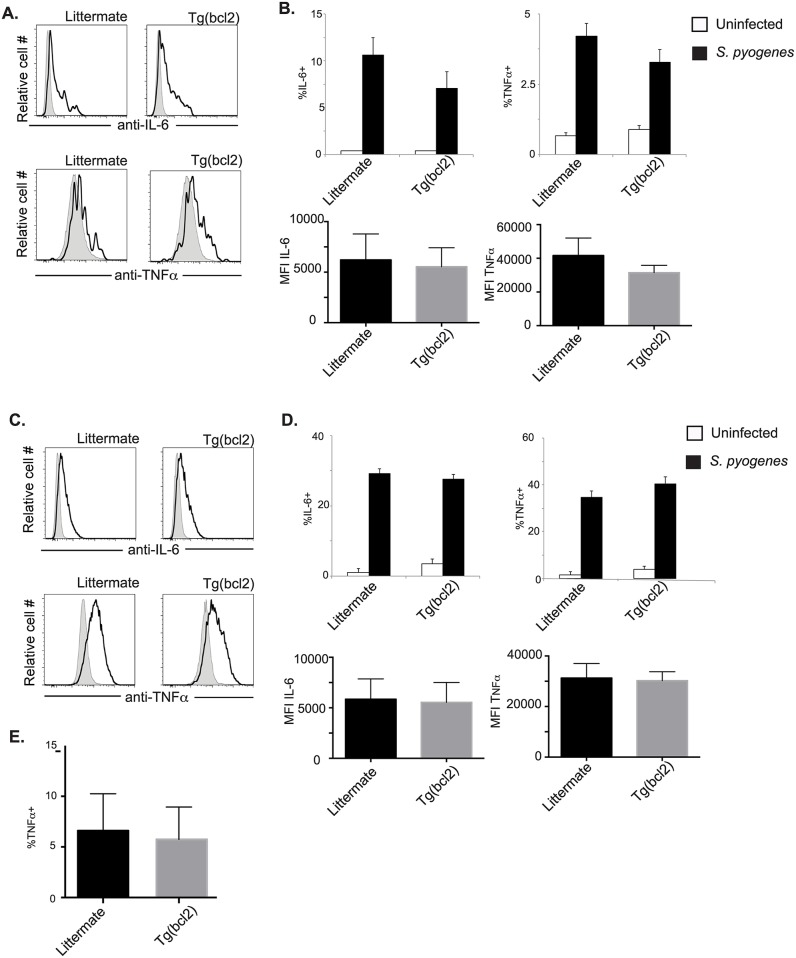
Cytokine production from myeloid cells after infection with *S*. *pyogenes*. Production of IL-6 and TNF**α** was measured by intracellular staining. Grey shaded histograms are uninfected and *S*. *pyogenes* infected are depicted with solid black lines. Representative staining of DCs is shown in (A), and the percent of cells expressing cytokines is shown in (B top), and the MFI is shown in (B bottom). Representative staining of macrophages is shown in (C), and the percent of cells expressing cytokines is shown in (D top), and the MFI is shown in (D bottom). Ex vivo isolated macrophages from the spleens of CD68(bcl2)tg and littermate mice infected with *S*. *pyogenes* were stained intracellular for TNF**α** (E). These data are representative of at least 3 independent experiments with n = 3–4 per experiment. The mean values are displayed. * denotes P value ≤ 0.05.

It seems likely that the increased systemic cytokine production in transgenic mice was due to the increased cellularity of infected mice. However, it is also possible that myeloid cells from CD68(bcl2)tg mice produce more cytokines. To investigate these non-mutually exclusive possibilities DCs and macrophages were stained intracellularly for IL-6 and TNFα. There was not a significant increase in the percentage of DCs making either cytokine when CD68(bcl2)tg mice were compared to littermate controls infected with *S*. *pyogenes* (Fig 15A and 15B (top)). Also the MFI was the same in DCs from transgenic animals or littermate controls (Fig 15B (bottom)), indicating that on a per cell basis the cytokine production is the same. Similar results were observed for macrophages. Cytokine production was equivalent between macrophages from transgenic mice and littermate controls, on a per cell and population basis (Fig 15C and 15D).

## Discussion

Pathogen clearance and host resilience are both important in surviving a given infection. While many studies have examined different mechanisms of pathogen clearance, recent studies have highlighted the importance that host resilience plays in survival of a given infection [2] [3] [4] [5]. The fact that cells of the myeloid lineage play important roles in both of these important processes [6–9] [10] [8,11–13] [14] [15] [16], we sought to determine how manipulation of myeloid cell death influenced the response to two bacterial pathogens. To do this we developed a mouse model that has decreased myeloid PCD. Ectopic expression of bcl-2 decreased PCD in response to numerous stimuli in myeloid cells of these mice (Figs 2, 3, 5, 9 and 13) [27]. We used two pathogens that interact with myeloid cells, but infect different areas of the host. The first pathogen, *L*. *pneumophila*, infects lung macrophages and remains confined to the lung [45,47,48] [46,75], while the second pathogen *S*. *pyogenes* spreads systemically [67] [35] [68] [69]. When myeloid cell death is prevented during systemic infection with *S*. *pyogenes* there is a significant decrease in host resilience (Figs 11, 12 and 14). Infected transgenic mice have decreased survival compared to littermate controls, and increased systemic inflammation. There is not a significant increase in bacterial load in the transgenic mice. The effects of decreased cell death during a pulmonary infection with *L*. *pneumophila* on host resilience are milder, but there is also an increase in inflammation in the lung. This increased inflammation precedes the small increase in bacterial load that is seen at the later stages of infection. Unlike infection with *S*. *pyogenes* both littermate and transgenic mice survive infection with *L*. *pneumophila*, indicating that the myeloid cell death has a greater impact on the systemic infection with *S*. *pyogenes* than on the pulmonary infection with *L*. *pneumophila*. In both infection models the primary cell type that is increased are neutrophils, which are known to cause tissue damage [34]. Also in both infection models the increase in inflammatory cytokine levels appears to be linked to the increased number of immune cells, and not by increased cytokine production on a per cell basis.

The lung is a delicate and essential organ thus the response to lung infections is particularly challenging, in that pathogen clearance must be balanced with host resilience mechanisms. As *L*. *pneumophila* is confined to the lung, we used this as a model of lung infection. Decreased cell death of lung myeloid cells leads to increased inflammation and immune cells infiltrate into the lung in response to this pathogen. Interestingly, this increased inflammation precedes any increase in bacterial load in the lung. There is a small, but statistically significant increase in the bacterial load in the lungs of transgenic mice compared to littermate controls, however surprisingly the decreased myeloid cell death did not lead to systemic spread of the bacteria. Transgenic mice had a decrease in health status compared to littermate controls as indicated by rapid and sustained weight loss. However, both genotypes of mice were able to survive and eventually clear the infection. The mild effect of decreased myeloid cell death observed upon infection with *L*. *pneumophila* may be for several reasons. Many different types of cell death are caused by infection with *L*. *pneumophila*, including pyroptotic cell death. Transgenic expression of bcl-2 has limited influence on pyroptotic cell death. However, the apoptotic cell death observed in later stages of infection of macrophages and also seen in DCs is profoundly affected (Fig 3) [27]. It could also be that the lung is able to cope using alternative resilience mechanisms, such as tissue repair pathways, with a threshold level of inflammation. It is likely, given the fragility and importance of the lung that there are multiple pathways that serve to protect this organ from damage. The increase in neutrophils observed in the transgenic mice could be a cause for the decreased health status, but pro-resilience pathways are able to maintain tolerance to the infection and survival is not impacted.

In order to determine what role myeloid cell death plays in pathogen clearance and host resilience during an infection that can spread systemically we infected mice with *S*. *pyogenes*. In our model this subcutaneous infection rapidly spreads systemically. This enabled us to examine what role myeloid cell death plays at the site of infection, in spread of the infection, and in the systemic response to infection. In contrast to what was observed after lung infection with *L*. *pneumophila*, where both genotypes were able to survive the infection, transgenic mice infected with *S*. *pyogenes* had significantly decreased survival compared to littermate controls. Interestingly, the decrease in myeloid cell death in the transgenic mice did not significantly influence pathogen spread and clearance. However, it did cause a significant increase in inflammation both at the site of the infection, and systemically. There was also an increase in liver damage in transgenic mice. These data suggest host resilience to systemic *S*. *pyogenes* infection is compromised by decreased myeloid cell death due to an excessive inflammatory response. During infection with *S*. *pyogenes* the bacteria spread systemically and the increase in inflammation at multiple sites may overwhelm other host resilience mechanisms. This indicates that PCD of myeloid cells is an essential disease resilience mechanism during systemic infections with *S*. *pyogenes*.

This study provides new insight into the roles that myeloid cell death plays in response to bacterial infections. While the decreased myeloid cell death caused minor inflammation in the lung, most likely other pathways were able to compensate for the increase in inflammation and prevent a large decrease in host resilience. However, during a systemic infection decreased PCD and consequently increased inflammation overwhelms the host and leads to decreased resilience as measured by survival. Interestingly, in both infections the main cell type that is increased are neutrophils. This increase may be caused either directly by the expression of bcl-2 in these cells or by the increase in other myeloid cell types that recruits neutrophils to the sites of infection. Neutrophils are known to cause damage in many different disease models [76–79], and they are the probable cause of the decreased host resilience with these infection models. As neutrophils are also essential for pathogen clearance depletion of them could result in decreased host-mediated damage, but the increase in bacteria will most likely cause an increase in pathogen-mediated damage. These data demonstrate how tightly regulated PCD in myeloid cells is, and how important it is for host resilience. Disruption of this regulation changes an infection that is potentially survivable to one that has a high rate of lethality. These findings can be applied to models of sepsis and other infections where host resilience processes are important factors in survival [67, 80].

## Materials and Methods

### Mice

A transgenic construct was made using the CD68 promoter and regulatory sequences as described by Gough et al. [81] and human bcl-2 cDNA was cloned into the XbaI restriction digest sites. The transgenic animals were made using standard methods. Four initial founders were selected based on screening by Southern Blot. The line used in this study Tg535 (bcl2)rm had the highest expression based on intracellular staining for the bcl-2 protein. The mice were initially on a 129/J background, but were backcrossed greater than 20 times to C57BL/6J mice. Animals were bred and maintained in a specific pathogen free facility (SPF)

### Ethics statement

All procedural protocols involving mice were approved by the appropriate Institutional Animal Care and Use Committee at the site where the work was performed. In Vienna all of the experiments have been approved by the Vienna University of Veterinary Medicine institutional ethics committee and performed according to protocols approved by the Austrian law called BMWF 68.205/0032-WF/II/3b/2014. General condition and behavior of the animals during the experiments was controlled by FELASA B degree holding personnel. The animal protocol number approved by University of Veterinary Medicine institutional ethics committee on 2/28/11 is 535233. Brown University adheres to the “U.S. Government Principles for the Utilization and Care of Vertebrate Animals Used in Testing, Research, and Training”, “PHS Policy on Humane Care and Use of Laboratory Animals”, “USDA: Animal Welfare Act & Regulations”, and “the Guide for the Care and Use of Laboratory Animals”. The University is accredited by the Association for Assessment and Accreditation of Laboratory Animal Care International (AAALAC). Brown University’s PHS Assurance Number: A3284-01 and it expires July 1, 2018 The USDA Registration Number is 15-R-0003. Brown University IACUC approved on October 8, 2013, and the animal protocol number is 1308000011.

### Bacterial strains and infection

The *L*. *pneumophila* strain used in this study was JR32ΔflaA provided by Craig Roy (Yale University School of Medicine, New Haven USA). The S. *pyogenes* strain used in this study was serotype M1 strains ISS3348 [82]. For *L*. *pneumophila* infections mice were infected intranasally with 1X10^6^ *L*. *pneumophila* in 40 μl sterile saline. Bacteria were grown from a heavy patch overnight in autolyzed yeast extract broth. For *S*. *pyogenes* infections mice were infected with 1X10^7^ bacteria in 50 μl subcutaneously in the left flank. A single colony of S. *pyogenes* was grown overnight in Todd Hewitt Broth (THB). This overnight culture was diluted into 100 ml of THB and grown for approximately 6 hours until in the log phase. Bacterial amounts were estimated using OD600 readings, and confirmed by colony forming unit (CFU) quantification. To ensure proper infectivity of cells when using the non-motile strain of *L*. *pneumophila*, bacteria were inoculated using a spinfection protocol. Bacteria were added to the cells in antibiotic-free media, and plates were spin for 5 minutes at 1200 rpm (290 RCF).

### Bacterial quantification

To determine the inoculation load and the bacterial loads in various organs at indicated time points after infection CFU assays were done. At several time points after *L*. *pneumophila* infection lungs, spleens, and livers from infected mice were homogenized in 2 ml of sterile water using an electronic homogenizer (Polytron 2100). One-hundred μl of serially diluted homogenate was plated on charcoal yeast extract (CYE) plates. Colonies were quantified after 2–3 days of incubation at 37 degrees C. To determine bacterial counts in skin and organs from mice infected with *S*. *pyogenes*, organs were homogenized in PBS, as described for *L*. *pneumophila* infection. Serially diluted homogenate was plated on blood agar plates and incubated overnight at 37 degrees C. Bacterial colonies were quantified the next day.

### Cell isolation and counts

To collect bronchoalveolar lavage fluid (BALF), the trachea was exposed, and a flexible tube placed on a 23-gauge cannula was inserted into the trachea. The lung was rinsed with 1ml PBS using an attached syringe. The viability of the isolated cells was determined by Trypan blue exclusion, and the cells were counted in a hemacytometer. For isolation of cells from lungs, they were perfused with 20 ml of PBS. The lung tissue was diced into small pieces and incubated for 45min at 37 degrees C in 4ml of media containing collagenase and DNAse. Afterwards, digested lung tissue was made into a single cell suspension by passage through a cell strainer. After centrifugation the cells were re-suspended in 4ml 40% Percoll/Roswell Park Memorial Institute (RPMI) and carefully layered over 4ml of 80% Percoll/PBS. The formed gradient was centrifuged at RT for 20min at 652rcf (Eppendorf 5810R centrifuge) with minimal acceleration and deceleration. Cells assembled in the interphase were collected, and washed with 10ml RPMI media containing 5% fetal calf serum.

### Flow cytometry staining

All steps of staining were performed on ice unless mentioned otherwise. All staining was done in V-bottom 96-well plates. Isolated cells were pelleted by centrifugation and washed twice with PBS containing 1% BSA and 0.001% w/v sodium azide (FB). Cells were re-suspended in FB containing rat anti-mouse CD16/CD32 antibodies (1:100) and incubated for 10min to block the Fc receptors, followed by two washes Cells were re-suspended in FB containing the desired antibodies for the surface staining in an appropriate dilution determined by titration. After 20min incubation, cells were washed twice with FB. Antibodies used included CD11c (M1/70) Biotin, Ly6C (AL-21) V450 (BD Biosciences), Ly6G (1A8) FITC, CD11c (N418) PE, F4/80 (BM8) APC, and 570 Fc receptor block (93) (Biolegend). In addition, the fixable viability dye eFluor 780 (eBioscience) (room temperature staining) and Streptavidin Brilliant Violet were used. Apoptotic cells were detected using either Annexin V (eBioscience) and PI (Sigma), or the CellEvent reagent and sytox (Thermofisher). To identify specific cell types these stains were combined with the cell surface markers described above. Stained cells were acquired on a FACSAria III cell sorter, equipped with a 488nm (blue) laser, a 633nm (red) laser, and a 407nm (violet) laser. Flow-cytometry was also performed on the Attune NxT. Finally, collected data were analyzed by FlowJo Software (Tree Star, Inc).

### Histology staining and scoring

For histological analysis, perfused lungs or excised skin were placed in 1% paraformaldehyde (PFA) overnight at 4 degrees C. The samples were transferred into 70% ethanol and the samples were processed in the Excelsior tissue processor. The samples were embedded in paraffin blocks and 5 μMsections were made with a microtome (Leica). Standard staining protocols were used for Hematoxylin and Eosin staining of rehydrated sections. All histological samples were assessed twice in a blinded manner. To determine the histological damage score (HDS), the following criteria were considered: the frequency (none = 0; sporadic = 0.5; few = 1; many = 2, excessive = 3) of neutrophils, macrophages and lymphocytes and their location (perivascular, peribrochial, parenchymal, subpleural, and alveolar lumen), as well as the activation of the pleura pulmonalis (none = 0; little = 0.5; medium = 1; strong = 2, excessive = 3). The points were added together and a maximum of 48 points per sample could be achieved. To assign the samples into 10 HDS groups (0–1 = 1; 1.1–2 = 2; 2.1–3 = 3; 3.1–4 = 4; 4.1–5 = 5; 5.1–6 = 6; 6.1–7 = 7; 7.1–8 = 8; 8.1–9 = 9; 9.1–10 = 10), the obtained sum was divided by 4.8. A low HDS value indicates minor tissue damage, whereas high HDS values mark increased damage of the lung. In addition, also the frequency of neutrophils, macrophages, and lymphocytes as well as the level of pleura activation was analyzed separately. Skin sections were also scored in a blinded manner.

### Quantification of cytokines, chemokines, and Alanine Transaminase (ALT)

To determine the cytokine and chemokine protein levels, the Mouse Th1/Th2/Th17/Th22 13plex FlowCytomix Multiplex assay was performed according to the manufacturer’s instructions. RNA from tissues was purified using Reliaprep RNA Miniprep System (Promega). Quantitative PCR was performed on a Roche LC96 using standard methods. Alanine transaminase in the serum was measured with the use of a colorimetric kit (Cayman Chemical) according to manufacturer’s instructions.

## Supporting Information

S1 Fig Bacterial CFUs systemically and day 10 after infection. There are no detectable *L*. *pneumophila* in the spleens, livers, or kidneys in either littermate or CD68(bcl2)tg mice 96 hours after infection (A). There are no detectable *L*. *pneumophila* in lungs 10 days after infection in either littermate or CD68(bcl2)tg mice (B). Data is from 3 independent experiments with n = 3–4 per experiment.
(TIF)
