# Correction: The Influence of Programmed Cell Death in Myeloid Cells on Host Resilience to Infection with *Legionella pneumophila* or *Streptococcus pyogenes*

**DOI:** 10.1371/journal.ppat.1006591

**Published:** 2017-08-30

**Authors:** Pia Gamradt, Yun Xu, Nina Gratz, Kellyanne Duncan, Lester Kobzik, Sandra Högler, Pavel Kovarik, Thomas Decker, Amanda M. Jamieson

There are multiple errors in the manuscript.

There is an error in reference 65. The correct reference is: “Speir M, Lawlor KE, Glaser SP, Abraham G, Chow S, Vogrin A, et al. Nat Microbiol. 2016 Feb 24;1:15034. doi: 10.1038/nmicrobiol.2015.34.”

The following information is missing from the Funding section: “Research reported in this publication was supported by an Institutional Development Award (IDeA) from the National Institute of General Medical Sciences of the National Institutes of Health under grant number P20GM103652.”
