# Supplementary figures and images for: The Influence of Programmed Cell Death in Myeloid Cells on Host Resilience to Infection with Legionella pneumophila or Streptococcus pyogenes
Source: PLoS Pathog. 2016 Dec 14;12(12):e1006032. doi: 10.1371/journal.ppat.1006032 (PMC5156374; doi:10.1371/journal.ppat.1006032)

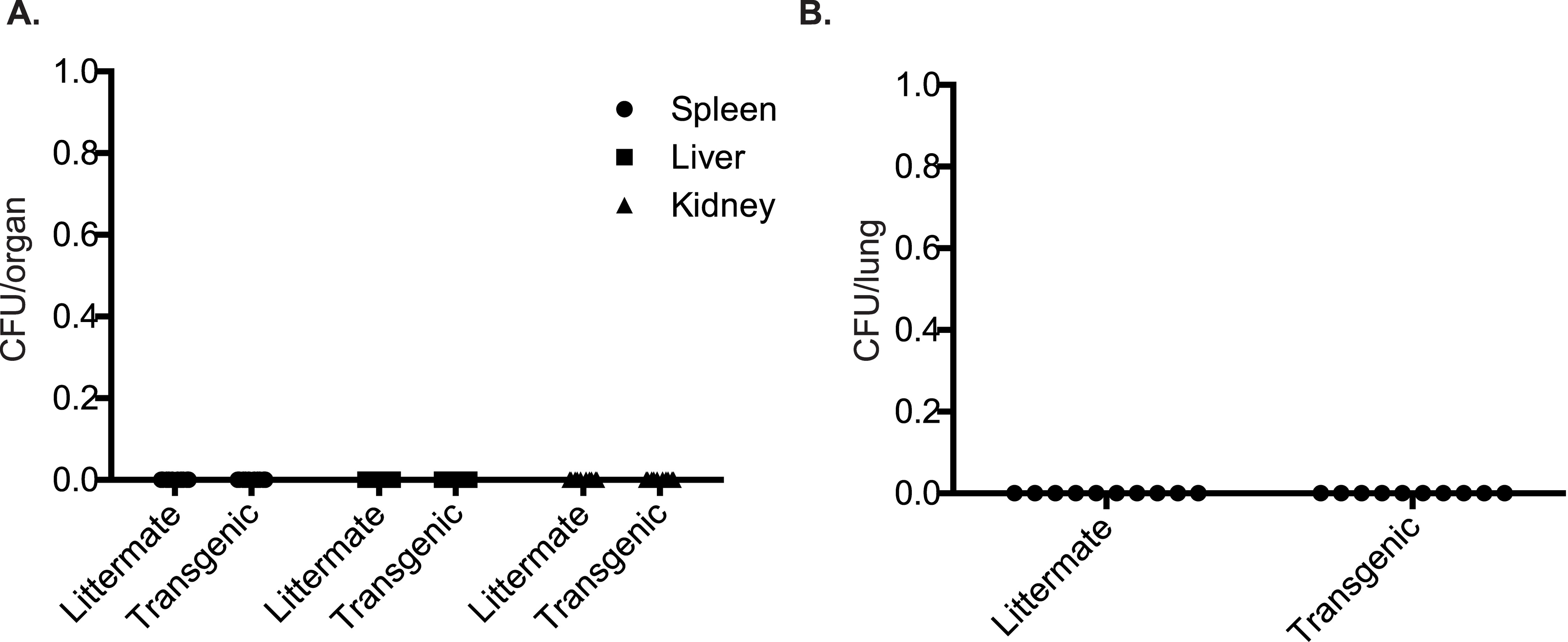

Supplement: S1 Fig — There are no detectable L. pneumophila in the spleens, livers, or kidneys in either littermate or CD68(bcl2)tg mice 96 hours after infection (A). There are no detectable L. pneumophila in lungs 10 days after infection in either littermate or CD68(bcl2)tg mice (B). Data is from 3 independent experiments with n = 3–4 per experiment. (TIF) [file ppat.1006032.s001.tif]
